# Bilateral Necrotic Foot Lesions of Kaposi Sarcoma in a Man from Ecuador

**DOI:** 10.4269/ajtmh.25-0755

**Published:** 2026-03-19

**Authors:** Su M. Aye, Nathan Asher, Glen Blackman, Stephen L. Walker

**Affiliations:** ^1^ Department of Dermatology, University College London Hospitals NHS Foundation Trust, London, United Kingdom;; ^2^ Department of Pathology, University College London Hospitals NHS Foundation Trust, London, United Kingdom;; ^3^ Department of Clinical Oncology, Macmillan Cancer Centre, University College London Hospitals NHS Foundation Trust, London, United Kingdom;; ^4^ Hospital for Tropical Diseases, University College London Hospitals NHS Foundation Trust, London, United Kingdom;; ^5^ Faculty of Infectious and Tropical Diseases, London School of Hygiene & Tropical Medicine, London, United Kingdom

**Figure 1.**
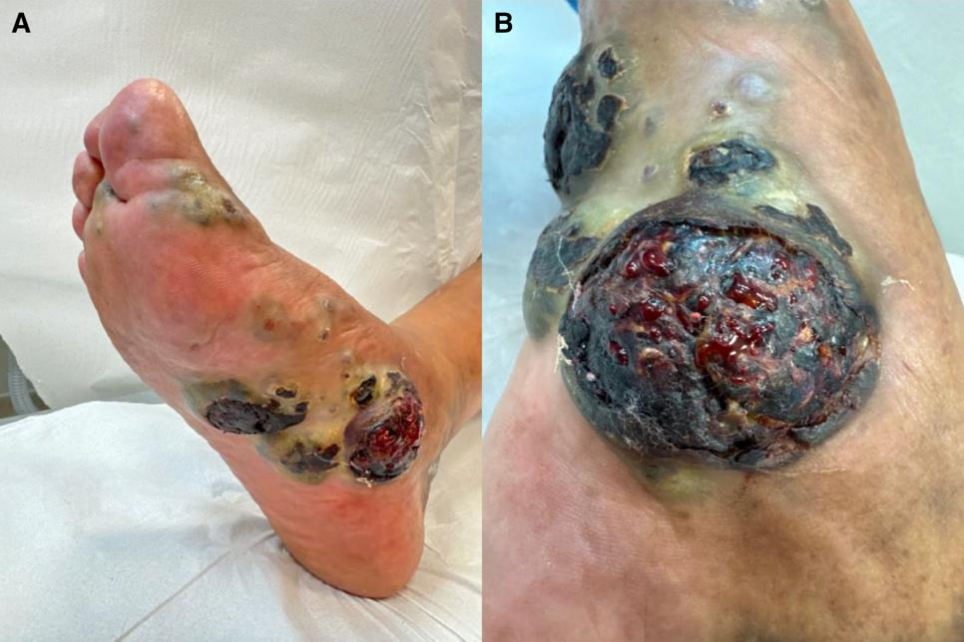
(**A**) Clinical presentation of the right foot showing multiple, painful, black, fungating, necrotic, ulcerated lesions (medial aspect [5 × 5 cm] and plantar surface [5 × 3 and 3 × 2.5 cm]). (**B**) Close-up view of the largest lesion demonstrating extensive necrosis with active bleeding from the ulcerated tissue.

A 77-year-old Ecuadorian man living in the United Kingdom for 5 years presented to emergency services. He stated that he had returned from a 2-year stay in rural Cotopaxi Province, Ecuador with progressively enlarging, painful, necrotic lesions on both feet (Figure 1). The patient noticed papular lesions 6 months earlier that had enlarged such that walking was very painful. He denied fever, gastrointestinal symptoms, or respiratory symptoms. He had had barefoot exposure to soil while engaged in farm work in Ecuador. Examination revealed multiple, firm, ulcerated, malodorous black nodules and plaques on both feet and associated right lower limb edema. Several papules were noted on the pinnae. There was no oral involvement, hepatosplenomegaly, or lymphadenopathy. Pedal pulses and sensory examination were normal. The differential diagnoses included paracoccidioidomycosis, cryptococcosis, Kaposi sarcoma (KS), and melanoma.

**Figure 2.**
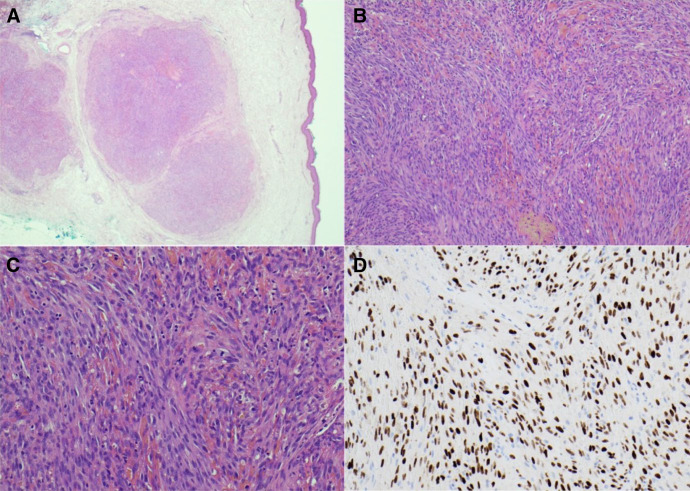
(**A**) Hematoxylin–eosin stain at 2× magnification showing a multinodular dermal tumor without connection to the overlying epidermis. (**B**) Hematoxylin–eosin stain at 10× magnification demonstrating a spindle-cell tumor with a vasoformative pattern and areas of intralesional hemorrhage. (**C**) Hematoxylin–eosin stain at 20× magnification showing slit-like vascular spaces admixed with inflammatory cells, hemorrhage, and mitotic figures. (**D**) Human herpesvirus-8 immunostaining at 20× magnification revealing speckled nuclear positivity in a subset of lesional cells.

Punch biopsies demonstrated a dermal spindle-cell tumor with a vasoformative pattern, intralesional hemorrhage, slit-like vascular spaces admixed with inflammatory cells, and mitotic figures characteristic of KS and confirmed by speckled nuclear human herpesvirus-8 (HHV-8) staining (Figure 2). Gram, diastase, periodic acid–Schiff, and Grocott stains were negative. Fungal and mycobacterial cultures of skin were negative. Galactomannan antigen in blood was negative, and beta-D-glucan serology was equivocal. Serum HHV-8 DNA was present in the blood but below the limit of quantification. Human immunodeficiency virus and human T-lymphotrophic virus serologies were negative.

Classical KS was diagnosed. Thorax, abdomen, and pelvis computed tomography scans did not demonstrate any extracutaneous disease. Intravenous liposomal doxorubicin was commenced, with marked regression of lesions and improved mobility after eight cycles.

Kaposi sarcoma is a rare, malignant vascular neoplasm of lymphatic endothelial origin induced by HHV-8, primarily affecting skin and mucous membranes.^1^ The five subtypes are classic (sporadic), endemic, iatrogenic immunosuppression associated, human immunodeficiency virus (HIV) associated, and KS in men who have sex with men without HIV infection.^1^ Clinical presentation varies from asymptomatic pink macules or papules to exophytic, ulcerated nodules with edema.^2^

Incidence of KS reflects rates of HHV-8 seropositivity in the population. HHV-8 seropositivity rates vary widely, with rates of over 40% in Africa south of the Sahara and less than 10% in Asia, northern Europe, and the United States.^3^ In South America, HHV-8 antibody prevalence is low among blood donors in Chile (3%), Argentina (4%), and Brazil (2.8–7.4%) but much higher among Amerindian populations in Ecuador and Brazil.^4^^,^^5^ The number of new cases in Ecuador in 2020 was estimated to be 56 (27.8–113.0), with higher estimates for Venezuela, Argentina, Peru, Colombia, and Brazil.^3^
